# Effect of Personal Exposure to PM_2.5_ on Respiratory Health in a Mexican Panel of Patients with COPD

**DOI:** 10.3390/ijerph120910635

**Published:** 2015-08-28

**Authors:** Marlene Cortez-Lugo, Matiana Ramírez-Aguilar, Raúl Sansores-Martínez, Rogelio Pérez-Padilla, Alejandra Ramírez-Venegas, Albino Barraza-Villarreal

**Affiliations:** 1 Instituto Nacional de Salud Pública, Morelos, Av. Universidad #655, Col. Santa María Ahuacatitlán, C.P. 62100 Cuernavaca, Morelos, México; E-Mail: cmarlene@insp.mx; 2 Comisión Federal para la Protección contra Riesgos Sanitarios, Monterrey #33, Col. Roma, Del. Cuauhtémoc, C.P. 06700 México, D.F., México; E-Mail: mramireza@cofepris.gob.mx; 3 Instituto Nacional de Enfermedades Respiratorias, Calz. Tlalpan #4502, Col. Sección XVI, Del. Tlalpan, C.P. 14080 México, D.F., México; E-Mails: perezpad@servidor.unam.mx (R.P.-P.); raulsansores@yahoo.com.mx (R.S.-M.); aleravas@hotmail.com (A.R.-V.)

**Keywords:** PM_2.5_ personal exposure, COPD, PEF

## Abstract

*Background*: Air pollution is a problem, especially in developing countries. We examined the association between personal exposure to particulate matter with an aerodynamic diameter less than 2.5 µm (PM_2.5_) on respiratory health in a group of adults with chronic obstructive pulmonary disease (COPD). *Methods*: All participants resided in Mexico City and during follow-up, personal exposure to PM_2.5_, respiratory symptoms, medications, and daily activity were registered daily. Peak expiratory flow (PEF) was measured twice daily, from February through December, 2000, in 29 adults with moderate, severe, and very severe COPD. PEF changes were estimated for each 10 µg/m^3^ increment of PM_2.5_, adjustment for severity of COPD, minimum temperature, and day of the sampling. *Results*: For a 10-µg/m^3^ increase in the daily average of a two-day personal exposure to PM_2.5_, there was a significant 33% increase in cough (95% CI, range, 5‒69%), and 23% in phlegm (95% CI, range, 2‒54%), a reduction of the PEF average in the morning of −1.4 L/min. (95% CI , range, −2.8 to −0.04), and at night of −3.0 L/min (95% CI, range, −5.7 to −0.3), respectively. *Conclusions*: Exposure to PM_2.5_ was associated with reductions in PEF and increased respiratory symptoms in adults with COPD. The PEF reduction was observed both at morning and at night.

## 1. Introduction

Short-term environmental exposure to particulate matter has been associated with adverse health effects, such as decrease in pulmonary function, increase in respiratory symptoms, hospitalization, and mortality [1,2,3,4,5,6,7,8,9,10,11,12]. Many of these studies have used the information of particles and other pollutants from fixed-site outdoor measurements for exposure assessment.

Air pollutants have also been associated with a decrease in lung function, measured by Peak expiratory flow (PEF) in children with asthma and in healthy children [13,14,15,16,17,18,19] and in healthy or susceptible adults [20,21,22,23].

On various occasions, series of studies have reported the effects of air pollution on lung function and respiratory symptoms in children. However, the few studies that have been published on adults with asthma [24,25], on individuals with and without chronic respiratory symptoms, and on adults with chronic obstructive pulmonary disease (COPD) [26,27,28] show inconsistent results of the effects of air pollution. On the other hand, COPD comprises an important cause of morbidity and mortality worldwide. While active cigarette smoking is the most important preventable risk factor globally, outdoor and indoor air pollutants can cause or exacerbate COPD. Studies during the last 20 years continue to show increased risk associated mainly with particulate matters, even at much lower levels [29].

The objective of this prospective cohort study was to determine the association between personal particulate matter with an aerodynamic diameter less than 2.5 µm (PM_2.5_) exposures and a decrease in lung function and respiratory symptoms in a cohort of adults with COPD residing in Mexico City.

## 2. Experimental Section

### 2.1. Participants

For the present analysis, we included 29 adults with COPD, all >40 years of age, and all treated at the National Institute of Respiratory Diseases (the INER) in Mexico City, a referral center for respiratory diseases where treatment is provided mainly for patients lacking health insurance. Details of data collection have been described previously [30]. Briefly, during the initial visit, study participants responded to questionnaires regarding past medical history, use of COPD medication, respiratory symptoms, household characteristics, and particle exposure. During follow-up, the study subjects were divided into six groups according to their place of residence; each group continuously carried personal monitors during a close to two-week period (12 days). This procedure was repeated three times throughout the year. Each 12-day period was denominated Phase 1, and 2, and 3. During follow-up, personal exposure to PM_2.5_, PEF, respiratory symptoms, medications, and activities were registered daily. All procedures were explained to the participants, who signed an informed consent form, and the study protocol was approved by the Ethical Committee of the Instituto Nacional de Salud Pública (National Institute of Public Health, INSP).

At the introductory meeting, participants were provided with an electronic PEF meter (Air Watch #001; Imetrikus, CA, USA), which can record up to 5000 tests, including measurements with the day and time: all participants were taught how to measure PEF according to international standards [31].

PEF was measured with the subject in a standing position, twice daily in the morning as soon as the participant got up, and at night before the subject went to bed; three peak-flow maneuvers were requested, and only the highest of the three was registered. Participants also recorded, in a personal diary, their symptoms (cough, wheeze, cold/influenza, phlegm, *etc.*) and their activities (including any passive smoking) in the different microenvironments and the use of medications. The fieldworker visited the participants each morning in order to check PEF measurements (taken from the PEF memory), to change personal filters, to review the participants’ recording of their activities of the previous day, and the daily signs, symptoms, and medications reported in the participant’s diary.

Personal pumps [30] with 37-mm Teflon filters (Whatman, Hillsboro, OR, USA) using a flow of 4 L/min were employed for personal samplers, which were attached to the shoulder strap of a bag (where the participants carried their personal pump and batteries); the filter’s impactor was situated near the subject’s breathing zone.

Details of personal monitoring equipment for PM_2.5_, quality control, and quality assurance procedures have been described previously [32].

### 2.2. Statistical Analysis

In order to analyze the effects of personal PM_2.5_ exposure concentrations on the PEF measured (highest value of the three performed maneuvers), we utilized the generalized estimating equation (GEE) [33] with a continuous response for longitudinal date and exchangeable covariance structure, a multivariate analog of linear regression to account for the within-participant correlation of the repeated measures. The models were run to evaluate exposure effects on the same day and for up to six previous days (lag 0 to 6).That is, the association was calculated by selecting the day that PEF measures were taken and by assigning the measurement of personal exposure to PM_2.5_ that same day (lag 0), considering the PM_2.5_ 24-h average. Covariates in the final models included COPD severity as follows: (Moderate: 50% ≤ Forced expiratory volume at 1 sec (FEV1) <80% predicted; Severe: 30% ≤ FEV1 < 50% predicted; Very severe: FEV_1_ < 30% predicted [34], and minimum temperature on the day that the PM_2.5_ sample was taken. Units of personal exposure were expressed in mass per unit volume (µg/m^3^), and PEF measurements were expressed in volume per time (L/min.).

In order to standardize the distribution of PEF values, each PEF value was transformed into a Z-score by subtracting the mean PEF of each participant and dividing the result by the standard deviation (SD) of each participant’s PEF values of each participant [35,36]. To report the results, we used the original PEF L/min measure. Models were adjusted for both the generalized estimating equation (GEE)_S_ and for mixed generalized model; we decided to use the GEE_S_ model with binomial family and logit link for greater simplicity because it considers only fixed effects. Moreover, the effects estimated between GEE and the mixed generalized model did not present a significant variation.

PEF circadian variability was calculated as (PEF at night-PEF at morning)/mean PEF.

Prevalence of respiratory symptoms was calculated for each day of the study by employing the day as unit of analysis, and symptoms were registered as presence or absence of symptoms (1 = yes; 0 = no). The effects of personal PM_2.5_ exposure concentrations on symptoms (overall symptoms and specific symptoms) were evaluated using GEE, with a binary outcome. These associations were explored and analyzed for the same day and for up to six previous days (lag 0 to 6) utilizing random effect models for repeated measurements with the logistic link (xtlogit). Models were adjusted for potential confounding factors, including COPD severity, previous-day minimum temperature, and chronological time.

All of the analyses were performed using the Stata statistical software package (Stata software ver. 9; Stata Corp., College Station, TX, USA).

**Table 1:** Participant characteristics by chronic obstructive pulmonary disease (COPD) severity status.

Variables	Moderate	Severe	Very Severe	Total
Subjects, *n*	7	13	9	29
Male subjects *n* (%)	3 (10)	9 (31)	8 (28)	20 (69)
Mean age (years) (range)	70 (63–76)	71 (62–80)	66 (43–72)	69 (43–80)
FEV1 % PREDICTED ^*^	59	35	23	37
Time indoors % (range)	87 (57–100)	90 (54–100)	93 (38–100)	90 (38–100)
PEF total *n*	219	447	320	986
Mean morning PEF (L/min ± SD)	242. ± 116	186 ± 98	160 ± 73	189 ± 99
Mean night PEF (L/min ± SD)	267 ± 139	183 ± 95	153 ± 73	192 ± 105
Mean PEF variability (%)	25.0	24.3	17.9	22.4
Frequency of medication use (%) ^†^	131 (84)	340 (96)	241 (96)	712 (94)
PM_2.5_ personal total *n*	80	163	122	365
Mean and range of *n* measurements in individuals	11 (7–16)	13 (5–22)	13 (7–20)	12 (5–22)
Mean personal PM_2.5_ (µg/m^3^ ± SD)	44 ± 25.4	36 ± 20	39 ± 21.9	39 ± 22.1

^*^ Moderate COPD: 50% ≤Forced expiratory volume at 1 sec (FEV1) <80% predicted; Severe: 30% ≤ FEV1 < 50% predicted; Very severe: FEV_1_ <30% predicted; ^†^ Bronchodilator, corticoids, and/or antihistamines. SD = Standard deviation; PEF = Peak expiratory flow.

## 3. Results and Discussion

**Figure 1:**
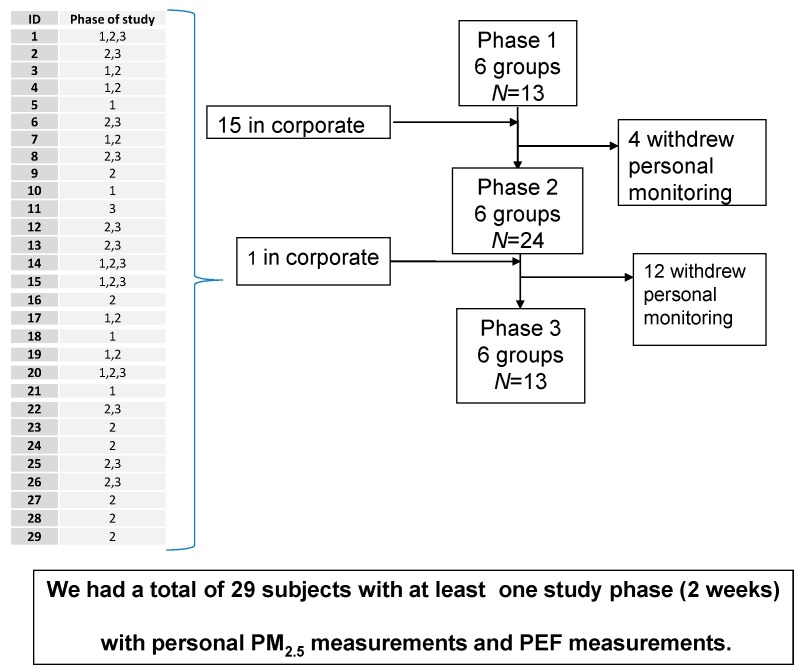
Personal exposure follow-up.

A total of 29 adults, nine female and 20 male subjects, participated in the study. Their age range was 43‒80 years, and mean age was 69 years. According to the classification of COPD severity recommended by Global Initiative for Lung Disease (GOLD) [34], seven participants were classified as having moderate or stage II COPD (26.7%), 13 as having severe or stage III COPD (43.3%), and nine as having very severe or stage IV COPD (30%). Based on the daily activity questionnaire, participants spent 90% of their time indoors during the study period (Table 1). Of the 29 participants, four completed three personal-monitoring phases, 14 completed two phases, and 11, only one phase. Between the first and second monitoring Phases 1 and 2, four subjects withdrew from the study and 15 were newly recruited in order to compensate for the loss of study subjects. Each study phase consisted of two weeks (12 consecutive days). However, personal monitoring entailed several problems with both the equipment and with the participants. Thus, it was not possible to meet this goal and we did not have the same number of PEF as Personal Monitors. The number and flow of subjects during the personal monitoring phases are depicted in Figure 1.

### 3.1. PEF, Symptoms, and Medication Frequency

The mean of morning PEF was 195 L/min (range, 51–626 L/min), and mean night PEF was 203 L/min (range, 51–669 L/min). PEF morning and night measurements were compared by analyzing their averages (Student *t* test), when it was noted that there was no significant difference between them (*p* = 0.1418). The average number of morning PEF measurements per participant was 23 (738 measurements in total), and the average of night PEF measurements per participant was 20 (592 measurements in total). Participants with moderate COPD reported less medication use (antihistamines, bronchodilators, and/or steroids) as compared with individuals with severe and very severe COPD (Table 1).

All patients had a heavy exposure to tobacco and fulfilled the criteria of post bronchodilator airflow obstruction; in addition lacked atopy, reported allergies and clinical features of bronchial asthma. Nevertheless patients had a significant circadian variability in PEF that overlaps considerably with that observed in asthma, similar to overlapping acute response to bronchodilators. We attempted to avoid this discussion by selecting patients with a heavy risk factor for COPD (smoking), but excluding patients with some reversibility to bronchodilators would also be arbitrary and would tend to reduce the real responses to air pollution that affect typical patients with COPD in their daily lives.

Throughout the study, there were 336 events of difficult breathing (39.3%), 278 events of cough with phlegm (32.5%), 253 events of cough (29.6%), 35 events of cold (4.1%), and 10 events of fever (1.2%) (Table 2).

**Table 2:** Prevalence of symptoms by chronic obstructive pulmonary disease (COPD) severity status during the study period.

COPD															
	Moderate				Severe				Very severe				Total		
	*n* = 121				*n* = 256				*n* = 178				*n* = 535		
Symptom	No.	%	X (range) ^*^		No.	%	X (range) ^*^		No.	%	X (range) ^*^		No.	%	X (range) ^*^
Wheeze	29	24	4 (0–9)		100	39	8 (0–35)		74	42	8 (0–27)		203	37	7 (0–35)
Phlegm	38	31	5 (0–21)		109	43	8 (0–35)		29	16	3 (0–12)		176	32	6 (0–35)
Cough	36	30	5 (0–18)		102	40	8 (0–35)		33	19	4 (0–12)		171	31	6 (0–35)
Cold/influenza	6	5	0.9 (0–5)		9	4	0.7 (0–3)		13	7	1 (0–7)		28	5	1 (0–7)
Fever	2	2	0.3 (0–2)		1	0.4	0.1 (0–1)		1	0.6	0.1 (0–1)		4	0.7	0.1 (0–2)

*n* = Number of days measured; No. = Number of events; ^*^ Mean and range of events among individuals.

### 3.2. Personal Exposure to PM_2.5_

**Figure 2:**
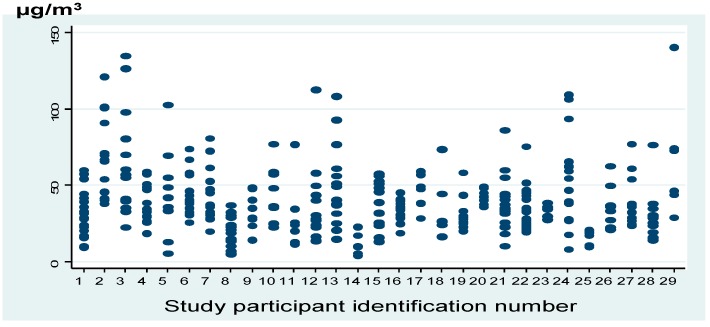
Distribution of Particulate matter with an aerodynamic diameter less than 2.5 µm (PM_2.5_) personal exposure according to individuals participating in the study, Mexico City.

Figure 2 illustrates variability by participant of personal exposure to PM_2.5_ considering internal and external sources. Daily average concentrations of personal PM_2.5_ were 38.4 µg/m^3^, and the 5th, 50th and 90th percentiles were 10.9, 34.0, and 66.0 µg/m^3^ during the study period, respectively.

### 3.3. Personal PM_2.5_ Effects on Health Outcomes

Statistical significant associations between personal PM_2.5_ and PEF or respiratory symptoms were observed at different day lags. An increase of 10 µg/m^3^ in personal PM_2.5_ levels was associated with a decrease in the morning PEF average of −1.4 L/min. (95% Confidence interval, 95% CI = −2.8 to −0.04) and in the night PEF average of −3.0 L/min (95% CI = −5.7 to −0.3) with a 2-day lag, respectively (Table 3). With a third-day lag, the decrement was only in the night PEF average deviation of −3.6 L/min. (95% CI: −6.5 to −0.7). Mean PEF variability was lower in very severe COPD**.** The effect of particles on the PEF mainly reflected in the 2- and 3-day exposure, which can be explained by time to develop inflammatory reaction by the increase in PM_2.5_.

**Table 3:** Association of peak expiratory flow (PEF) and personal particulate matter with an aerodynamic diameter less than 2.5 µm (PM_2.5_), Mexico City.

Personal PM_2.5_	Change ^a^ Morning PEF L/min	95% CI ^b^	Change Night PEF L/min	95% CI	PEF Varia-Bility (%)	95% CI
Lag 0	−0.71	−2.2–0.77	0.008	−2.1–2.1	0.4	−0.5–1.3
Lag 1	0.01	−1.4–1.4	0.16	−2.3–2.6	0.2	−0.8–1.3
Lag 2	−1.4	−2.8–−0.04 ^*^	−3.0	−5.7 to −0.3 ^*^	0.7	−0.5 to 1.9
Lag 3	−1.2	−2.9 to 0.43	−3.6	−6.5 to −0.7 ^*^	0.7	−0.6 to 2.0
Lag 4	−1.2	−3.1 to 0.67	−0.9	−4.3 to 2.5	−0.4	−1.8 to 1.0

**^a^** Estimated using Generalized estimating equation (GEE) models and adjusted for Chronic obstructive pulmonary disease (COPD), minimum temperature, and chronological time. Decrease in PEF per 10 µg/m^3^ change in personal PM_2.5._; **^b^** Confidence interval;^*^ p < 0.05.

**Figure 3:**
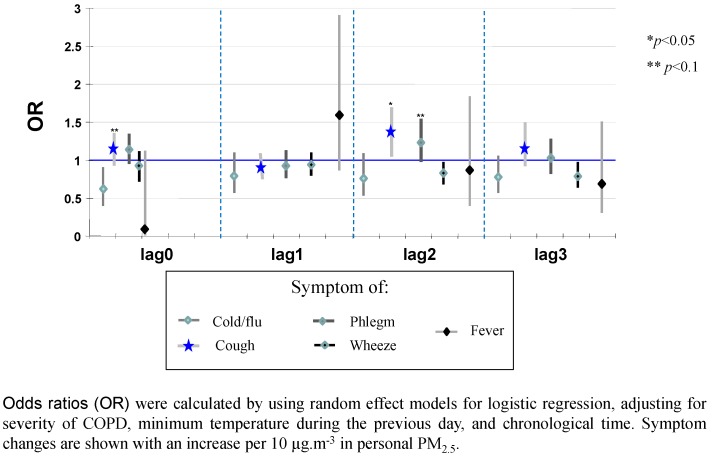
Effect of personal of exposure to particulate matter with an aerodynamic diameter less than 2.5 µm (PM_2.5_) on respiratory symptoms, Mexico City.

There was significant and positive association between personal exposure to PM_2.5_ and respiratory symptoms such as cough and phlegm, adjusted for minimum temperature, the sampling day, or COPD severity. For each 10-µg/m^3^ increase of personal PM_2.5_, there was a 33% increase of cough (95% CI = 5%; 69%) and 23% in phlegm (95% CI = −2%; 54%) with a 2-day lag. Frequency of cough increased by 18% (95% CI = −2%; 41%) for each 10 µg/m^3^ increase in personal PM_2.5_ exposure on the same day. The remainder of the symptoms did not exhibit a positive association with exposure to PM_2.5_ (Figure 3).

The effect of particles PM_2.5_ on the symptoms, cough (significant) and phlegm (borderline) prove airway inflammation or irritation. With respect to other symptoms that were not statistically significant, this can be due to less important effects or effects that entertain a more limited relationship with the process of inflammation or irritation caused by the particles or by the lack of adjustment by other contaminants not measured in the study.

We observed that personal exposure to PM_2.5_ increased the occurrence of cough and phlegm in adults with COPD, with a maximum increase observed after a two-day lag. Impact on PEF measurements presented at both morning and night for a two-day lag, and the highest decrease was registered on night PEF on day 3 prior to exposure. The PEF decrease found in our study was higher than that of several published studies in which exposures of the subjects were either fixed monitoring stations or monitors inside or outside the subject’s home. Recent studies have shown that particulate exposure includes the combination of particles from the combustion of fixed and mobile sources or secondary sources of sulfates, as well as various kinds of materials related with personal activity [37,38,39]. The results reported in this study are consistent with those of several studies that have shown an association between exposure to fine particulate matter and a decrease in PEF, which peaks after a lag of between one and five days [16,39,40,41].

A limitation of our study lies in the fact that we did not measure other contaminants were not counted with measurements that could have affected the respiratory function, mainly the metals contained in the particulate material, zinc and iron, which have been associated with the decrease of pulmonary function in adults with COPD [1]. On the other hand, a strong point of the present study comprises the use of measured personal exposure to estimate the change associated with PEF and respiratory symptoms, which diminish the consequences of exposure classification errors of the subjects. Personal exposure measurements include not only contributions from outdoor environmental sources, but also indoor contributions, mobility patterns, and personal activities, which vary during the day and from person to person. The information provided supports efforts to reduce air pollution and its impact on susceptible individuals, such as patients with COPD. 

The subjects of this study spent the majority of their day indoors; this, domestic sources reported previously (pets, mold, cooking, aerosol use) likely played a very important role in personal exposure [32]. Smoking inside the home and the use of carpets (in the house) are also significant domestic sources of PM_2.5_ [32,42,43]. In this study, despite the fact that some relatives of some of the participants reported smoking in the home, passive smoking was not a significant source in the model of personal exposure to PM_2.5_ or in the model for a decrease in PEF.

Some potential aspects for explaining the association found between short-term exposure to PM_2.5_ and lung health in this population are the following: PM_2.5_ represents the aerodynamic diameter less than 2.5 µm fraction of particulate matter and increases and sustains oxidative stress (OS) both on the entire respiratory tract and at the systemic level, where OS induces inflammation. In the lungs, PM may cause inflammation, thereby aggravating an underlying lung disease, thus reducing the efficacy of lung-defense mechanisms, and animal studies have shown increased vulnerability to PM in animal with cardiopulmonary disease [44].

Also, patients with COPD often have bronchial hyper reactivity that, in several studies, has been associated with exposure to air pollutants [45,46]. As our data demonstrate, exposure to fine particles that penetrate into the deepest areas of the lung is associated with lung symptoms and altered lung function in patients with COPD. This is important information that can aid in establishing protective and preventive measures specific for this type of susceptible population in urban areas with conditions similar to those of Mexico City.

Reducing the risk from indoor and outdoor air pollution is feasible and requires a combination of public policy and protective steps taken by individual patients. Public policy directed toward reducing vehicle and industrial emissions to safe levels is an urgent priority in order to reduce the development of COPD symptoms, as well as exacerbations in, and the hospitalization of, those with COPD disease which is a significant source of poor quality of life (QoL), mortality, and high treatment costs.

## 4. Conclusions

In conclusion, exposure to particulate matter with an aerodynamic diameter less than 2.5 µm (PM_2.5_) is associated with increased respiratory symptoms in patients with COPD and a decrease in lung function. Also, the results reported in this study suggest that repeated peak flow measurements in patients with COPD provide a helpful tool able to identify adverse environmental impacts; these measurements should include spirometry and potentially more sensitive markers for inflammation pathways in areas such as exhaled Nitric oxide (NO), Interleukin (Il)-8, or another inflammatory cytokine [47,48,49,50].
